# Comparison of Weight-Gain-Based Prediction Models for Retinopathy of Prematurity in an Australian Population

**DOI:** 10.1155/2023/8406287

**Published:** 2023-08-17

**Authors:** Alexander Bremner, Li Yen Chan, Courtney Jones, Shaheen P. Shah

**Affiliations:** ^1^University of Sydney, Ophthalmology, Camperdown 2006, NSW, Australia; ^2^Mater Mother's Hospital Brisbane, Raymond Tce, South Brisbane 4101, QLD, Australia; ^3^University of Queensland, Ophthalmology, Woolloongabba 4102, QLD, Australia

## Abstract

**Purpose:**

Four weight-gain-based algorithms are compared for the prediction of type 1 ROP in an Australian cohort: the weight, insulin-like growth factor, neonatal retinopathy of prematurity (WINROP) algorithm, the Children's Hospital of Philadelphia Retinopathy of Prematurity (CHOPROP), the Colorado Retinopathy of Prematurity (CO-ROP) algorithm, and the postnatal growth, retinopathy of prematurity (G-ROP) algorithm.

**Methods:**

A four-year retrospective cohort analysis of infants screened for ROP in a tertiary neonatal intensive care unit in Brisbane, Australia. The main outcome measures were sensitivities, specificities, and positive and negative predictive values.

**Results:**

531 infants were included (mean gestational age 28 + 3). 24 infants (4.5%) developed type 1 ROP. The sensitivities, specificities, and negative predictive values, respectively, for type 1 ROP (95% confidence intervals) were for WINROP 83.3% (61.1–93.3%), 52.3% (47.8–56.7%), and 98.4% (96.1–99.4%); for CHOPROP 100% (86.2–100%), 46.0% (41.7–50,3%), and 100% (98.4–100%); for CO-ROP 100% (86.2–100%), 32.0% (28.0%–36.1%), and 100% (98.3–100%); and for G-ROP 100% (86.2–100%), 28.2% (24.5–32.3%), and 100% (97.4–100%). Of the five infants with persistent nontype 1 ROP that underwent treatment, only CO-ROP was able to successfully identify all.

**Conclusions:**

CHOPROP, CO-ROP, and G-ROP performed well in this Australian population. CHOPROP, CO-ROP, and G-ROP would reduce the number of infants requiring examinations by 43.9%, 30.5%, and 26.9%, respectively, compared to current ROP screening guidelines. Weight-gain-based algorithms would be a useful adjunct to the current ROP screening.

## 1. Introduction

Retinopathy of prematurity (ROP), a disease of the developing retinal vasculature of premature infants [1, 2], is a significant cause of adverse events and morbidity such as retinal detachment and irreversible visual impairment [1, 2].

Infants at risk of developing ROP undergo repeated retinal screening examinations to detect severe disease that requires treatment [3–5]. Current ROP screening guidelines recommend examination of infants below a certain gestational age (GA) and birth weight (BW) which are determined according to the local characteristics of the premature population and the quality of neonatal care [6] (e.g., GA less than 31 weeks and BW less than 1250 g in Queensland, Australia). Infants with higher GA or BW than screening cutoffs who have an unstable clinical course are also screened by the judgement of the neonatologist [7].

The detection yield of ROP screening is low. According to several years of the Australian and New Zealand Neonatal Network Annual Reports [8], it is clear that the significant majority of infants screened for ROP have a low likelihood of developing severe disease (3-4%) [8], and this matches studies in other developed nations [9–13]. Current screening methods for ROP cause neonatal distress including hypertension, decreased oxygen saturation, and the oculocardiac reflex [4, 14, 15]. Other issues with the low detection yield of ROP screening include parental anxiety [4] and frequent hospital presentations or prolonged hospital admissions for screening [16]. As only a small number of infants examined require treatment for ROP, improving the detection of ROP has the potential to increase the cost-effectiveness of current ROP screening [7].

With a greater understanding of the pathophysiology of ROP, clinical studies have shown that prolonged early IGF-1 deficits are associated with a higher risk of subsequent sight-threatening ROP [1, 2, 17]. Deficiencies in IGF-1 lead to a hypoxic preclinical phase resulting in a more severe subsequent proliferative vascular clinical phase. However, as routine serial IGF-1 level monitoring would be challenging to obtain and costly, postnatal weight gain has been adopted as a surrogate [10].

Several screening algorithms using postnatal weight gain to reflect serum IGF-1 levels have been developed in the last decade [9, 10, 18–21]. These algorithms utilise the postnatal weight gain in combination with the GA and BW characteristics to signal an alarm that a particular infant has a high risk of developing severe ROP. Reflecting the variable and complex nature of this disease, these algorithms must be validated at a local level prior to being implemented into clinical practice. Therefore, further studies in Australian cohorts are required [7].

To our knowledge, no studies have directly compared the outcomes of all four algorithms for the same cohort. This study aims to compare the performance of four algorithms, namely CHOP-ROP (Children's Hospital of Philadelphia ROP) [18, 19], WINROP (weight, insulin-like growth factor I, neonatal ROP) [10, 20], CO-ROP (Colorado retinopathy of prematurity model) [21], and G-ROP (postnatal growth and retinopathy of prematurity) [9] in an Australian tertiary level NICU setting. The secondary objective is to estimate the impact of a more targeted screening process in reducing the number of examinations in low-risk infants to focus on high-risk infants.

## 2. Methods

This retrospective cohort study was conducted from January 2017 to December 2020 including all premature infants admitted to the neonatal intensive care unit (NICU) at the Mater Hospital in Brisbane, Queensland, who underwent ROP screening (criteria GA < 31 and/or BW < 1250 g or who had an unstable clinical course determined by the treating neonatologist). Digital wide field images and standard binocular indirect ophthalmoscopy (if required) were used to diagnose and classify ROP.

Weight data were collected from a review of the electronic records. Infants with the following were excluded from this study: the presence of clinical conditions that cause nonphysiological weight gain (including hydrocephalus and severe subcutaneous oedema) and infants with incomplete medical records (for example, infants transferred from another hospital without weight gain data or infants who were having ongoing retinal screening but who deceased prior to the determination of final ROP outcome were excluded).

Data collected included birthweight, gestational age, ROP outcomes including treatment, treatment modality, the postmenstrual age at the time of treatment, and all postnatal weight gain measurements until discharge from the ROP screening clinic. The ETROP study [22] classification was the basis for the categorisation in our study (no ROP, mild ROP, type 1 ROP, and type 2 ROP). Mild ROP is the presence of ROP that does not meet the criteria for type 1 or type 2 ROP.

Data were entered into the following four weight gain predictive algorithms according to their inclusion criteria.

### 2.1. WINROP

Only infants less than 32 weeks of gestation at birth irrespective of the BW are eligible to be entered into the WINROP algorithm, which is available online [10, 20]. Birth weight, date of birth, gestational age, and weekly weights were entered until 40 weeks of postmenstrual age or discharge, or till the alarm signals in the algorithm, whichever was earlier. WINROP algorithm allows postnatal weight to be entered until 40 weeks of postmenstrual age (PMA) to classify risk.

### 2.2. CHOPROP

Infants less than 31 weeks of GA or less than 1501 g birthweight are eligible to be evaluated by CHOPROP. Birthweight, gestational age, and daily weight gain rate are entered into the algorithm to calculate the risk score from 2nd week onwards [19]. CHOPROP requires the documentation of neonatal weight at the end of the second week to be included in the algorithm. Weight change in the first week was disregarded as per the original study. The daily weight gain rate was calculated by weekly measurements (the difference between current weight and previous week's weight is divided by 7). Alarm cutoff of >/ = 0.014 was used to identify neonates at risk of type 1 ROP.

### 2.3. CO-ROP

Infants less than or equal to 30 weeks of GA and who have a birthweight of less than 1501 g are eligible to be evaluated by CO-ROP [21]. They also should not gain more than 650 g by the 28th day of life.

### 2.4. G-ROP

This is the latest of the algorithms to be designed and utilised by the largest cohort of infants during development [9]. Infants meet the criteria for ROP screening if they met any of the following six criteria: (1) birthweight <1051 g; (2) gestational age <28 weeks; (3) weight gain between day 10 and 19 <120 grams; (4) weight gain between day 20–29 <180 grams; (5) weight gain between day 30–39 <170 grams; (6) diagnosis of hydrocephalus. For infants not meeting the GA, BW, or hydrocephalus criteria for G-ROP, there was no weight measurement at a particular measurement day (for instance, day 10, 19, 20, 29, 30, and 39 for G-ROP), and then, the nearest weight measurement (within 2 days) was used.

Diagnostic performances of all four algorithms were described by calculating sensitivity, specificity, PPV, NPV, and likelihood ratios. The Wilson method was used to determine the 95% confidence intervals for all calculations. We also sought to calculate the efficiency of these algorithms by calculating the reduction in the number of infants that would require eye examinations. For this, we proposed that the algorithms were utilised to make decisions on whether infants would be screened or not based on whether they were alarmed or not. Infants that did not alarm would not undergo an eye examination.

Ethical approval for the study was obtained from the Mater Research Governance (HREC/15/MHS/112).

## 3. Results

**Figure 1:**
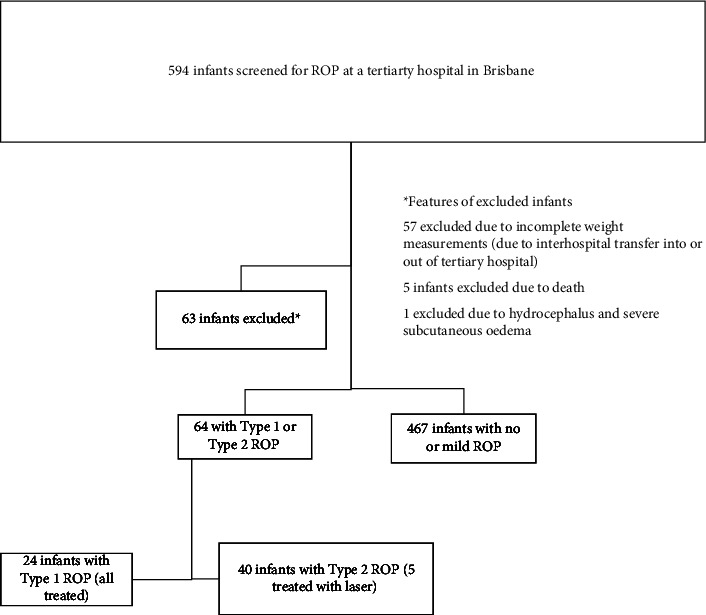
Flowchart for patients included in the validation comparison of weight-gain-based prediction models for retinopathy of prematurity in an Australian population.

Five hundred thirty-one infants met the inclusion criteria.  Figure 1 describes the study flow and reasons for exclusion from the study.

531 infants included in the study had a median BW of 1100 g (IQR 432 g) and the median GA of 28 weeks (IQR 3 weeks). Of the infants, 296 (55.7%) were male. Any ROP developed in 356 infants (67.0%), of whom 24 (4.5%) developed type 1 ROP, and all received intravitreal bevacizumab injections and/or laser retinal photocoagulation. A further 40 (7.5%) infants developed type 2 ROP of whom 5 received late laser retinal photocoagulation for nonresolving activity/ongoing ischaemia. The demographics of included infants are found in Table 1.

Diagnostic performances of all four screening algorithms are shown in Table 2. All 531 infants were entered into CHOP-ROP, CO-ROP, and G-ROP to determine the risk of type 1 ROP (Table 2). Using WINROP, a total of 508 infants were entered (the remaining 23 infants were more than 32 weeks gestation, and none of these 23 infants developed type 1 or type 2 ROP).

All four algorithms have high negative predictive values (NPV) for type 1, type 2, and treated ROP. CO-ROP had the highest negative predictive values and 95% confidence intervals (95% CI) for type 1, type 2, and treated ROP closely followed by G-ROP. The 95% CI for NPV for an infant with a negative test result on CO-ROP that does not have type 1 ROP, not have type 2 ROP, and not have treatment requiring ROP was 98.3–100%, 94.7%–99.4%, and 97.7–100%, respectively. Likelihood ratios for all algorithms screening type 1, type 2, treated, and any ROP were calculated and included in Table 2. We found that the negative likelihood ratios were consistent with the high negative predictive values for type 1 ROP and treated ROP.

If the algorithms were used to reduce the number of infants requiring examinations, G-ROP would have reduced the number of infants requiring examinations by 143 (26.9%) including 4 neonates with type 2 ROP infants of which one required treatment; compared to 162 (30.5%) for CO-ROP including 3 infants with type 2 ROP, none of whom required treatment; if CHOPROP was used 233 (43.9%), infants would not undergo examinations, and this number includes 8 infants with type 2 ROP of which 2 required treatment; and WINROP would reduce the number of infants undergoing examinations by 252 (49.7%) which includes 13 infants with type 2 ROP of which 1 required treatment and 4 infants with type 1 ROP (who all required treatment).

## 4. Discussion

We present, for the first time, a comparison of the sensitivities, specificities, predictive values, and likelihood ratios for multiple weight gain algorithms within a single Australian cohort.

Our study found a prevalence of 4.5% of type 1 ROP in infants screened for ROP between 2017 and 2020 which is similar to other prevalence studies in industrialised nations. Postnatal Growth and Retinopathy of Prematurity (G-ROP) retrospective cohort study conducted in 29 hospitals [11] found a prevalence rate of 6.1% of infants developing type 1 ROP. In a large Swedish cohort, the rate was 5.3% [12], and in a UK cohort, the prevalence rate was 4.0% [13].

In this study's Australian cohort, WINROP had the lowest sensitivity for detecting type 1 ROP. Of all the algorithms WINROP has been the most extensively studied; 36 studies across the world with a resultant range of sensitivities from 100% to 55% [23]. There is one other study conducted within an Australian population that found a sensitivity of 85.7%, a specificity of 59.0%, an PPV of 7.0%, and an NPV of 99.1% [24]. A recent systematic review found that WINROP has a sensitivity of 89%, specificity of 57%, and a negative likelihood ratio of 0.19 [23]. There were three validation studies that found sensitivities less than 75% [25–27]. Zepeda-Romero et al. argued that their low sensitivity was because the cohort of infants studied were exposed to unmonitored supplemental oxygen that caused larger and more mature infants to develop severe ROP [26]. When the validation study was repeated, with NICU changes implementing monitoring with constant pulse oximetry, oxygen saturation targets of 85–95%, alarms for oxygen saturation at 90–95%, and education courses for medical and nursing staff, the authors found an increased sensitivity of 80% [28]. A Taiwanese study also noted poor sensitivity in WINROP which was suggested to be a likely consequence of regional variation in expected weight gains between a South-East Asian premature infant and a European premature infant [27]. Models developed from small cohorts can be overfitted resulting in undesirable outcomes when validation studies in other regions of the world are performed [7]. Such results highlighted the need to perform such validation studies within our own neonatal population prior to implementation into clinical practice.

There are, in comparison, significantly fewer worldwide validation studies on CHOPROP, CO-ROP, and G-ROP [18–20, 23, 29–43], but, to date, the sensitivity results for type 1 ROP are promising (consistently between 90 and 100%). When assessed against the same cohort of 7483 infants that were part of developing the G-ROP model, CHOPROP had a sensitivity of 98.5% (95% confidence interval between 96.9 and 99.3%) [29] and CO-ROP had a sensitivity of 96.0% (95% confidence interval 93.4–97.6%) [32]. CO-ROP ranges from 93.1% to 100% sensitivity for type 1 ROP in American validation studies [20, 30–32], and CHOPROP ranges from 97.9% to 100% sensitivity for type 1 ROP in American, Italian, and Chinese validation studies [19, 29, 33, 34].

In 7 of 9 worldwide validation studies of G-ROP, the sensitivity for detecting type 1 ROP was 100% [9, 36–41], however in small cohort studies in Portugal and Turkey the sensitivity was 91% [42, 43]. A systematic review of postnatal weight gain algorithms has found that G-ROP has a worldwide sensitivity for type 1 ROP of 100%, specificity of 60%, negative likelihood ratio of 0.00, and positive likelihood ratio of 2.5 [23]. Our study had a significantly lower specificity compared to the systematic review (28.2% vs 60%). We take note of an outlier study by Caruggi et al. [39] that found a specificity of 100% which has skewed the specificity of Athikarisamy et al.'s meta-analysis [23]. If Caruggi et al.'s study [39] is excluded, the specificity ranges between 15% and 42%.

We found the algorithms did not have 100% sensitivity in detecting type 2 ROP; however, it should be noted that WINROP and CHOPROP were designed to identify type 1 ROP only. In our cohort, we found a prevalence of 4.7% of persistent nontype 1 ROP that required treatment. This is similar to Koucheki et al. [44] who found 4.9% of infants in the Canadian population. These rates are lower than that found by Liu et al. [45] who performed a secondary analysis of data from the G-ROP study to look at the prevalence and indications for treating infants who did not meet ETROP type 1 ROP criteria. They found that of the 1004 eyes of 514 infants who received treatment for ROP, 126 eyes of 91 infants (0.8% of all eyes and 12.5% of treated eyes in G-ROP) were treating nontype 1 ROP [45]. In Koucheki et al., the decision to treat the cases of nontype 1 ROP with unfavourable structural outcomes was made before postmenstrual age 45 [44], whereas in our study, this occurred from postmenstrual age week 45 and up to postmenstrual age week 55. Of the five infants with persistent nontype 1 ROP that underwent treatment, only CO-ROP was able to successfully identify all (CHOPROP alarmed 3 out of the 5 infants). As there are no clear current guidelines on whether to treat or observe persisting nontype 1 ROP, the clinical experience of the ophthalmologist and the patient factors ultimately determine management. This ultimately limits the utility of these algorithms in these neonates.

The use of weight-gain-based algorithms could be a useful adjunct to screening by reducing the number of infants requiring examinations. We found a reduction in eye examinations with the utilisation of weight-gain-based ROP algorithms ranging from 26.9% for G-ROP to 43.9% for CHOPROP. For CHOPROP, CO-ROP, and G-ROP, our study found similar percentages to other studies in the reduction of number of eye examinations needed [19, 20, 23, 29–35]. Our study and other worldwide studies show that weight-gain-based ROP algorithms would help reduce the workload for ROP screening by better targeting premature infants most at risk for developing severe ROP.

To date, validation studies have predominantly only reported sensitivities and specificities and have not included other evaluations of screening tests such as predictive values and likelihood ratios [9, 10, 18–21, 23–43]. These indicators do not allow an individual clinician to safely decide whether a particular infant can be safely excluded from screening, especially if the infant has a high pretest probability of developing severe ROP (such as extremely preterm or extremely low birthweight/adverse clinical course). We report on likelihood ratios as they can be a better way to apply the results of diagnostic tests to the individual patient [46].

There are some limitations in this study. Due to its retrospective nature, there was no method to standardise the data collection; for example, a difference of 1 day in the weight gain measurement can modify the alarm thresholds (particularly, for WINROP and CHOPROP). In addition, 63 infants out of 594 (10.6%) infants were excluded predominantly because there were not enough postnatal weight measurements to be able to determine an alarm risk. This is a limitation as some algorithms require longitudinal weight measurements and infants who have had their neonatal care in a different hospital and subsequently get transferred do not have complete postnatal weight gain data. This data loss has been a noted issue previously [25]. Furthermore, retrospectively assessing clinical records limits the ability to accurately differentiate physiological from nonphysiological weight gains.

A new ROP predictive model has been proposed known as DIGIROP [47]. This model utilises data available at birth for greater convenience and was demonstrated to be as accurate as CHOPROP, WINROP, and CO-ROP [48]. Once the latest version becomes available, further validation studies would be useful.

Our findings suggest that weight-gain-based ROP predictive models could play a role as an adjunct to ROP screening and add to reported sensitivities and specificities worldwide. Weight-gain-based ROP predictive models would improve the balance between reducing screenings and ensuring timely intervention so that healthcare systems can allocate resources more efficiently. Caution should be advised if these algorithms are used as screening criteria, as 3 of the 4 algorithms missed at least one infant that required treatment. Weight-gain-based algorithms are also limited as around 10% of infants were excluded due to interhospital transfer, death, and nonphysiological weight gain.

The direction of future studies in weight-gain-based ROP predictive models should not only continue to study the efficacy of these algorithms in the local population but also report the negative predictive values and negative likelihood ratios in addition to sensitivity and specificity. Future studies could also assess how easily weight-gain-based algorithms can be incorporated into ROP screening clinics such as by measuring the time added to the clinic list preparation when utilising these algorithms. The cost-effectiveness of weight-gain-based ROP predictive models could be determined from the number of infants that would not require screening. Prospective studies should be considered to collect an increasing amount of data that can optimise the criteria that weight-gain-based ROP predictive models are based on. From these studies, it can be determined whether weight-gain-based ROP predictive models can shift the cutoff screening criteria for ophthalmoscopy screening to a lower gestational age and birth weight.

## Figures and Tables

**Table 1:** Demographics of infants included in the study.

Characteristics	No ROP (*N* = 175)	Mild ROP (*N* = 292)	Type 2 ROP (*n* = 40)	Type 1 ROP (*N* = 24)	Total (*N* = 531)
*Birth weight, grams*					
Mean (SD)	1257 (265)	1078 (301)	912.2 (248.0)	719.4 (201.8)	1107 (310)
Median (IQR)	1255 (393)	1050 (369)	883 (329)	656 (231)	1100 (432)
Range	610–2040	510–2600	475–1490	420–1220	420–2600
% <1000 g	18.9%	45.5%	62.5%	91.7%	40.1%
% ≥1000 g	81.1%	54.5%	37.5%	8.3%	59.9%

*Gestational age, weeks*					
Mean (SD)	29 + 6 (2)	28 + 0 (2)	27 + 0 (2)	25 + 0 (2)	28 + 3 (2)
Median (IQR range)	30 + 0 (2)	28 + 0 (3)	27 + 1 (3)	24 + 5 (3)	28 + 3 (3)
Range	25 + 2–34 + 3	23 + 1–37 + 1	23 + 4–31 + 2	23 + 0–27 + 5	23 + 0–37 + 1
% <28 weeks	12%	49.0%	67.5%	100%	40.5%
% ≥28 weeks	88%	51.0%	32.5%	0%	59.5%
Gender (female), no (%)	78 (44.6%)	132 (45.2%)	17 (42.5%)	6 (33.3%)	235 (44.3%)

**Table 2:** Predictions of type 1 ROP, type 2 ROP, and treated ROP for CHOPROP, WINROP, CO-ROP, and G-ROP.

	CHOPROP	WINROP	CO-ROP	G-ROP
Type 1 ROP	*n* = 24	*n* = 24	*n* = 24	*n* = 24
Sensitivity, % (95% CI)	100	83.3	100	100
	(86.2–100)	(64.1–93.3)	(86.2–100)	(86.2–100)
Specificity, % (95% CI)	46.0	52.3	32.0	28.2
	(41.7–50.3)	(47.8–56.7)	(28.0–36.1)	(24.5–32.3)
Positive predictive value, % (95% CI)	8.1	8.0	6.5	6.2
	(5.5–11.7)	(5.2–12.0)	(4.4–9.5)	(4.2–9.0)
Negative predictive value, % (95% CI)	100	98.4	100	100
	(98.4–100)	(96.1–99.4)	(98.3–100)	(97.4–100)
Positive likelihood ratio (95% CI)	1.85	1.75	1.47	1.39
	(1.64–2.00)	(1.43–2.14)	(1.60–1.94)	(1.26–1.48)
Negative likelihood ratio	0.00	0.32	0.00	0.00
	(0.00–0.69)	(0.13–0.78)	(0.00–0.72)	(0.00–1.13)
Type 2 ROP	*n* = 40	*n* = 40	*n* = 40	*n* = 40
Sensitivity (95% CI)	80.0	67.5	92.5	90.0
	(65.0–90.0)	(52.0–79.9)	(80.1–97.4)	(76.9–96.0)
Specificity (95% CI)	45.8	52.0	32.4	28.3
	(41.6–50.2)	(47.5–56.5)	(28.4–36.6)	(24.5–32.5)
Positive predictive value, % (95% CI)	10.7	10.6	10.0	9.3
	(7.7–14.8)	(7.4–15.0)	(7.4–13.5)	(6.8–12.6)
Negative predictive value, % (95% CI)	96.6	95.0	98.1	97.2
	(93.4–98.3)	(91.6–97.0)	(94.7–99.4)	(93.0–98.9)
Positive likelihood ratio	1.48	1.41	1.37	1.26
	(1.24–1.76)	(1.11–1.78)	(1.23–1.52)	(1.12–1.41)
Negative likelihood ratio	0.44	0.62	0.23	0.35
	(0.23–082)	(0.40–0.98)	(0.08–0.69)	(0.14–0.90)
Treated ROP	*n* = 29	*n* = 29	*n* = 29	*n* = 29
Sensitivity (95% CI)	93.1	82.8	100%	96.6
	(78.0–98.1)	(65.5–92.4)	(88.3–100)	(82.8–99.4)
Specificity (95% CI)	46.0	52.6	32.3	28.3
	(41.7–50.4)	(48.1–57.0)	(28.3–36.5)	(24.5–32.4)
Positive predictive value, % (95% CI)	9.1	9.6	7.9	7.2
	(6.3–12.9)	(6.5–13.8)	(5.5–11.1)	(5.0–10.2)
Negative predictive value, % (95% CI)	99.1	98.1	100	99.3
	(96.9–99.8)	(95.5–99.2)	(97.7–100.0)	(96.1–99.9)
Positive likelihood ratio (95% CI)	1.72	1.75	1.48	1.35
	(1.52–1.96)	(1.44–2.11)	(1.34–1.57)	(1.23–1.47)
Negative likelihood ratio	0.15	0.33	0.00	0.12
	(0.04–0.57)	(0.15–0.73)	(0.00–0.91)	(0.02–0.84)
Any ROP	*n* = 356	*n* = 350^†^	*n* = 356	*n* = 356
Sensitivity (95% CI)	69.9	54.9	78.4	78.4
	(65.0–74.5)	(49.6–60.0)	73.8–82.3)	(73.8–82.3)
Specificity (95% CI)	72.0	63.2	48.6	37.7
	(4.9–78.1)	(55.6–70.2)	41.3–55.9)	(30.9–45.1)
Positive predictive value, % (95% CI)	83.6	76.2	75.6	71.9
	(78.9–87.3)	(70.6–81.0)	(71.0–79.7)	(67.2–76.1)
Negative predictive value, % (95% CI)	54.1	39.5	52.5	46.2
	(47.7–60.4)	(33.7–45.5)	(44.8–60.0)	(3.82–54.3)
Positive likelihood ratio	2.50	1.49	1.52	1.26
	(1.95–3.20)	(1.19–1.86)	(1.31–1.78)	(1.11–1.43)
Negative likelihood ratio	0.42	0.71	0.45	0.57
	(0.35–0.50)	(0.61–0.84)	(0.35–0.57)	(0.44–0.75)

^†^Six infants GA >32 cannot be entered into WINROP.

## Data Availability

The data used to support the study can be requested from Mater Mother's Hospital Brisbane.
